# Synergistic performance evaluation of MoS_2_–hBN hybrid nanoparticles as a tribological additive in diesel-based engine oil

**DOI:** 10.1038/s41598-023-39216-0

**Published:** 2023-08-02

**Authors:** Thachnatharen Nagarajan, Nanthini Sridewi, Weng Pin Wong, Rashmi Walvekar, Virat Khanna, Mohammad Khalid

**Affiliations:** 1 Faculty of Defence Science and Technology, National Defence University of Malaysia, 57000 Kuala Lumpur, Malaysia; 2 Sunway Centre for Electrochemical Energy and Sustainable Technology (SCEEST), School of Engineering and Technology, Sunway University, 47500 Petaling Jaya, Selangor Malaysia; 3 School of Computer Science and Engineering, Taylor’s University, 47500 Subang Jaya, Selangor Malaysia; 4 Department of Mechanical Engineering, MAIT, Maharaja Agrasen University, Baddi, 174103 HP India; 5 Division of Research and Development, Lovely Professional University, Phagwara, 144411 Punjab India; 6 School of Engineering and Technology, Sharda University, Greater Noida, 201310 Uttar Pradesh India

**Keywords:** Energy science and technology, Engineering, Materials science

## Abstract

In this study, MoS_2_–hBN hybrid nanoparticles were synthesized using an advanced microwave platform for new nanolubricant formulations. The synthesized nanoparticles were characterized by field-emission scanning electron microscopy, energy-dispersive X-ray spectroscopy, X-ray diffraction, and Raman spectroscopy. The hybrid nanoparticles were then introduced into a 20W40 diesel-based engine oil to produce a nanolubricant. The physical and chemical properties of the nanolubricant were investigated, including the viscosity index, stability, volatility, tribological properties, oxidation properties, and thermal conductivity. The results showed that the inclusion of 0.05 wt% MoS_2_–hBN hybrid nanoparticles in the oil significantly reduced the coefficient of friction and wear scar diameter by 68.48% and 35.54%, respectively. Moreover, it exhibited substantial oxidation and thermal conductivity improvement of 38.76% and 28.30%, respectively, at 100 °C. These findings demonstrate the potential of MoS_2_-hBN hybrid nanoparticles as an effective additive to enhance the properties of nanolubricant significantly. Furthermore, this study offers valuable insights into the underlying mechanisms responsible for the observed enhancements. The promising outcomes of this investigation contribute to the advancement of nanotechnology-based lubricants, showcasing their potential for improving engine efficiency and prolonging the lifespan of machinery.

## Introduction

Transportation is a key contributor to global energy consumption and greenhouse gas (GHG) emissions, which drive climate change and global warming. A significant portion of the energy consumed in transportation is used to overcome friction and wear in a vehicle’s moving parts, resulting in significant energy losses and environmental impacts^1,2^. Therefore, it is crucial to reduce friction and ensure the mechanical elements of the systems are wear-resistant^3^. Lubrication plays a critical role in achieving these objectives by reducing friction and wear, conserving energy, reducing emissions, and prolonging the lifespan of components. In the presence of a lubricant, a sliding film forms, significantly reducing friction, wear, and tear between mating surfaces^4–6^. Machine lubrication is highly dependent on the tribological qualities of the lubricating media. However, traditional lubricants face limitations in meeting the increasing demands for high-performance lubrication under extreme conditions while also being environmentally friendly^7–9^.

Recent advances in nanomaterials have paved the way for developing nanolubricants engineered at the nanoscale to exhibit enhanced engine tribological performance, oil characteristics, and fuel economy^10,11^. Nanolubricants have emerged as a promising solution to address the sustainability challenges of transportation by reducing energy consumption, minimizing wear, and lowering emissions. To further enhance the tribological, thermal and oxidative properties of SAE 20W40 engine oil, this study investigates the synergistic effect of a hybrid of molybdenum disulphide (MoS_2_) and hexagonal boron nitride (hBN) nanoparticles as additives. By incorporating the MoS_2_–hBN hybrid, this study aims to improve engine oil performance. MoS_2_ is a highly effective nanomaterial for reducing friction and wear due to its excellent lubricating properties. As a result, it has become a popular additive in lubricants^12–14^. MoS_2_ nanoparticles have a hexagonal crystalline structure, and their intrinsic lubricity properties are due to the weak van der Waals forces between the S–Mo–S sandwich layers and the pure positive charge on the surface, which causes electrostatic repulsion to spread. This allows layers with weak molecular forces to readily glide on one another, reducing friction and wear on mating surfaces^15,16^.

Similarly, hBN is another commonly used nanomaterial for lubricant additives^17,18^. The hBN has several advantages, including the same crystalline structure as graphene, making it a “white graphene”^19,20^. The hBN is a graphite isomorph with boron and nitrogen atoms filling the non-interacting A and B sublattices in the Bernal structure. The excellent tribological behaviour of hBN arises from the strong in-plane ionic bonding of the planer hexagonal crystalline lattice. Additionally, hBN is highly inert and lacks dangling bonds or surface charge entrapment, further contributing to its exceptional properties^21,22^.

These hybrid nanoparticles were synthesized using an advanced microwave synthesis platform, significantly reducing synthesis time and energy consumption^23^. This work endeavors to shed light on the underlying mechanism by which the hybrid MoS_2_–hBN additive improves the behavior of the base oil. Parameters such as the coefficient of friction, wear scar diameter, oxidation induction time (OIT), and Noack volatility are being investigated to better understand the additive’s effects. The findings of this study will provide valuable insights into the performance enhancement of engine oil through the use of hybrid MoS_2_–hBN additives.

## Results and discussion

- Chemical and structural characterization of nanoparticle

**Figure 1:**
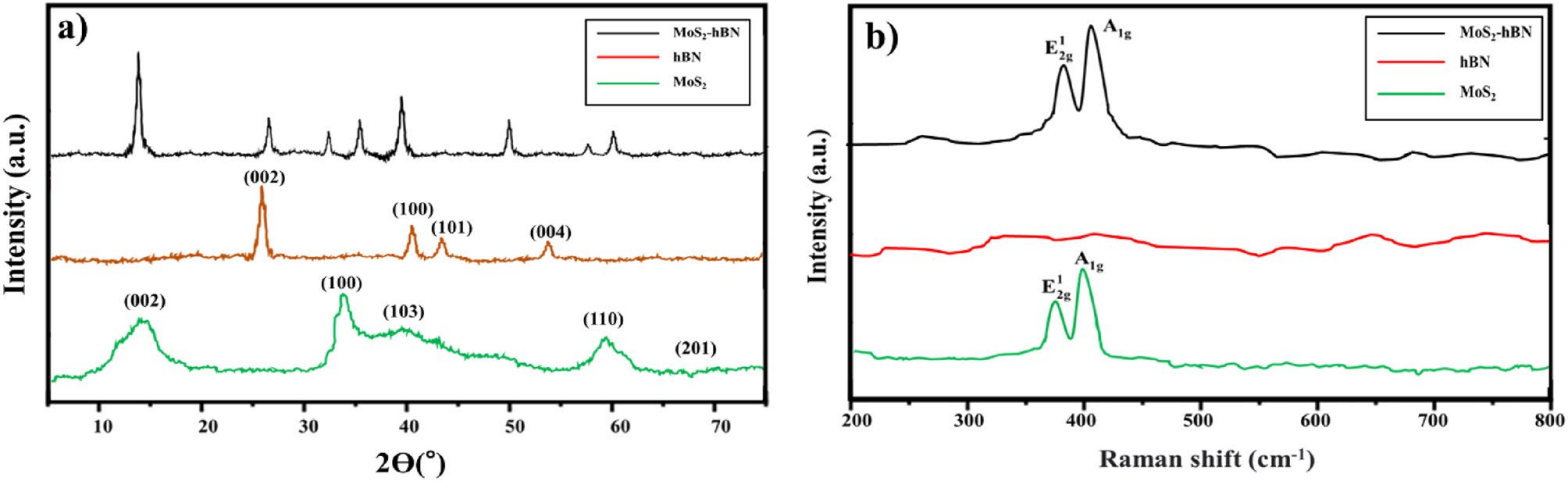
(**a**) XRD spectrum of the MoS_2_–hBN hybrid, hBN, and MoS_2_ nanoparticles. (**b**) Raman spectra of the MoS_2_–hBN hybrid, hBN, and MoS_2_ nanoparticles.

In Fig. 1, the XRD and Raman spectra were utilized to investigate the physiochemical characteristics of MoS_2_, hBN nanoparticles, and synthesized hybrid MoS_2_–hBN particles. According to the XRD analysis in Fig. 1a, the diffraction peaks at 2θ = 14.5°, 33.0°, 39.3°, 58.5°, and 69.7° are related to the (002), (100), (103), (110) and (201) peaks of the pure MoS_2_ phase (JCPDS no. 371492)^24,25^. The diffraction peaks of hBN are illustrated at 2θ = 27.4°, 42.7°, 43.6, and 54.3 peaks from the (002), (100), (101), and (004) planes^26,27^. The hybrid nanoparticle exhibited the same peaks linked with both nanoparticles, with no new peaks or significant shifts from the original MoS_2_ or hBN diffraction spectra. The Scherrer equation was used to estimate the crystallite size.

1 $$D=\frac{K\lambda }{\beta\;cos\theta }$$

In order to estimate the crystallite size of the MoS_2_–hBN hybrid nanostructure, Eq. (1) was used, where D represents the crystallite size in nanometers, K is the Scherrer constant set to 0.9, λ is the wavelength of X-rays, β is the full width at half maximum (FWHM), and θ is the peak position. The resulting value for the crystallite size of the MoS_2_–hBN hybrid nanostructure was 75.6 nm.

Figure 1b displays the Raman spectrum of the MoS_2_ nanosheets, which reveals the characteristic bands of this nanostructure. The first peak at 367 cm^−1^ is attributed to the E^1^_2g_ vibrational mode, while the second peak at 406 cm^−1^ is related to the A_1g_ mode. These modes correspond to in-plane vibrations of sulfur and molybdenum atoms in different directions and to out-of-plane vibrations (A_1g_) of sulfur atoms. An intense peak was observed for the sample containing hBN. However, hBN peaks were only slightly detected, possibly due to the limited penetration depth of the Raman laser on the surface of MoS_2_ nanoparticles above the hBN nanostructure caused by the encapsulation of MoS_2_ on the hBN surface.

**Figure 2:**
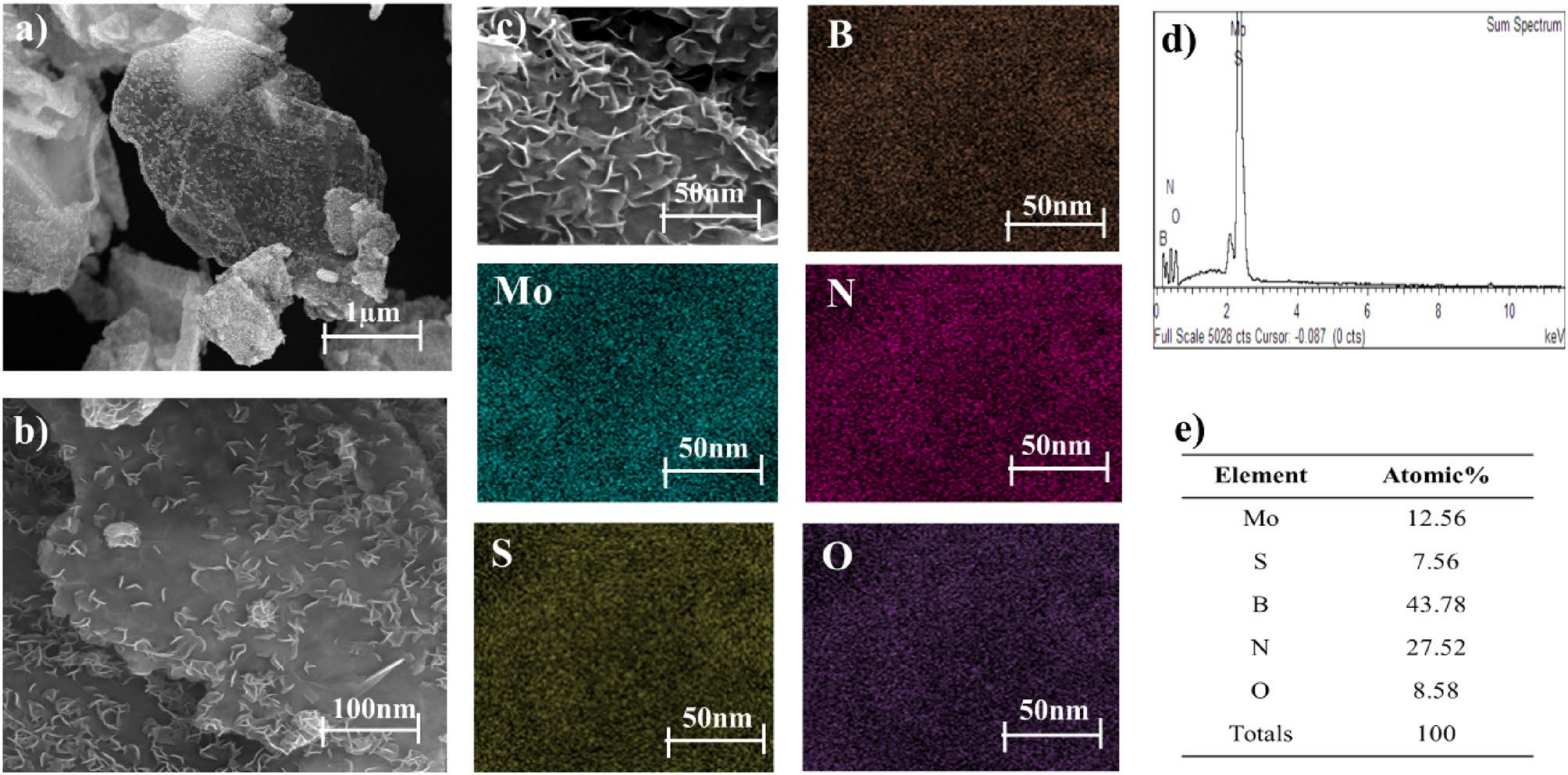
(**a**–**c**) FESEM image at three different magnification levels (Mo), (S), (B), (N) and (O) EDS Mapping composition (**d**) EDS spectrum (**e**) Elemental distribution of the MoS_2_–hBN hybrid nanoparticle.

Figure 2a–c presents SEM images at various magnifications, EDX spectrum, and elemental mapping of the hybrid MoS_2_–hBN nanoparticles. The images reveal that the nanoparticles have a uniform distribution with corrugated edges that are evenly faceted. Typically, the lateral size of hBN is smaller than that of MoS_2_, which can fill the gaps, potholes, and spaces between the MoS_2_ flakes, reducing the porosity and surface roughness of the hybrid nanoparticle. The size and thickness of the flakes range from 100 to 300 nm, confirming that they are in the nanometer range. This nanostructure offers a significant surface area-to-volume ratio for reactions to take place while minimizing the possibility of nanoparticle aggregation. Furthermore, during the advanced microwave synthesis, MoS_2_ encapsulates the hBN sheets’ surface and intercalates between the MoS_2_ layers, inducing interlayer coupling between the hybrid layers. Figure 2 illustrates the uniform and homogeneous distribution of Molybdenum (Mo), Sulphur (S), Boron (B), Oxygen (O), and Nitrogen (N) across the nanosheet via high-resolution EDS mapping composition. The EDS spectrum of the hybrid MoS_2_–hBN nanoparticles depicted in Fig. 2d confirms the existence of Mo, S, B, N, and O elements, while the corresponding quantitative surface analysis in Fig. 2e demonstrates the uniform elemental distribution of the respective elements in hybrid MoS_2_–hBN.

(b) Physiochemical characterization of nanolubricant.

The density, kinematic viscosity, and viscosity index of the nanolubricant with varying concentrations of MoS_2_–hBN hybrid nanoparticles are presented in Table 1. The results in Table 1 indicate that adding MoS_2_–hBN hybrid nanoparticles did not significantly affect the density of the oil. Engine oils must reduce their viscosity at low temperatures to reduce friction, improve fuel efficiency, and maintain a high enough viscosity at high temperatures to protect engine parts and ensure durability. This can be achieved by maintaining the viscosity at high temperatures, which contributes to the increase in the viscosity index of the engine oil^28,29^. The viscosity index (VI) is a measure of the change in an oil’s viscosity due to temperature changes, and it was calculated in this study using Eq. (2).

2 $${\text{VI}} = \frac{L - U }{{{\text{L}} - {\text{H}}}} \times 100$$

where U represents the oil’s kinematic viscosity at 40 °C, and L and H represent the reference oil’s kinematic viscosity at 40 °C and 100 °C, respectively, according to ASTM D2270.

**Table 1:** Density, kinematic viscosity and viscosity index of the nanolubricant with various concentrations of MoS_2_–hBN hybrid nanoparticle.

Sample name	Density 40 °C (g/cm^3^)	Kinematic viscosity (mm^2^/s)		Viscosity index
		40 °C	100 °C	
MoS_2_–hBN 0.005 wt%	0.8774	113.55	12.74	104.91
MoS_2_–hBN 0.01 wt%	0.8775	113.43	12.94	107.96
MoS_2_–hBN 0.05 wt%	0.8776	113.54	12.81	105.95
MoS_2_–hBN 0.1 wt%	0.8775	113.69	12.79	105.48
SAE 20W40	0.8770	113.76	12.73	104.53

Table 1 presents the density, kinematic viscosity, and viscosity index values of the nanolubricant with various concentrations of MoS_2_–hBN hybrid nanoparticles. The results indicate that there is a slight decrease in the kinematic viscosity of the oil after adding the nanoparticles. This reduction may be attributed to the lubricating effect of the nanoparticles, which can decrease the friction between oil molecules and moving parts, thereby lowering the resistance to flow. The hybrid nanoparticles can form a boundary layer between the engine components, reducing metal-to-metal contact and the energy required for movement at high shear rates^30–32^.

Furthermore, the addition of MoS_2_–hBN hybrid nanoparticles leads to an increase of 0.3–3.28% in the viscosity index of the engine oil. This is due to the nanoparticles’ ability to form a stable and uniform film on the moving components, even under high loads and temperatures, owing to their high shear strength. The formation of a protective layer on metal surfaces by the nanoparticles helps reduce friction and wear between the engine components, maintaining the oil’s viscosity at higher temperatures and increasing the viscosity index^13,33^. The MoS_2_–hBN hybrid nanoparticles can also enhance the thermal stability of the engine oil, preventing it from breaking down and thinning out at high temperatures, contributing to a higher viscosity index. Overall, the addition of MoS_2_–hBN hybrid nanoparticles can improve the performance of engine oils by reducing friction and wear, increasing thermal stability, and maintaining the viscosity index at high temperatures^34,35^.

**Figure 3:**
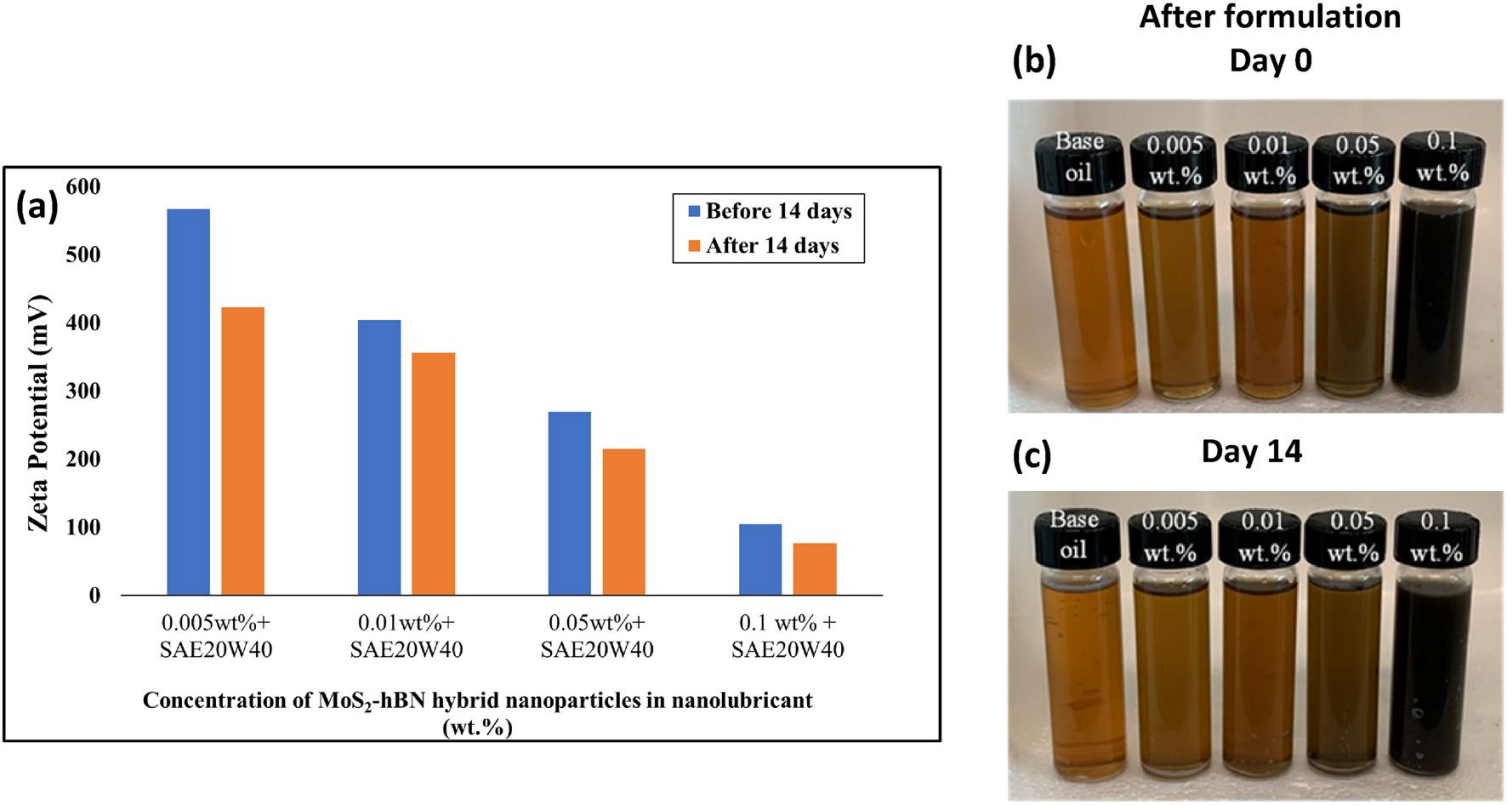
(**a**) Zeta potential values of nanolubricant with various concentrations of MoS_2_–hBN hybrid nanoparticle (**b**) stability after formulation, day 0 and (**c**) stability after day 14.

The zeta potential analysis of the MoS_2_–hBN hybrid based nanolubricant is carried out to measure the electrical charge of the nanoparticles in the engine oil, and it is an important parameter to evaluate the stability of the hybrid nanoparticles in the engine oil. This method to study the stability of the nanolubricant is widely used by researchers^36,37^. Figure 3 shows the zeta potential values of nanolubricant with different concentrations of MoS_2_–hBN hybrid nanoparticles before and after 14 days. The stability of MoS_2_–hBN hybrid nanolubricant was directly proportional to the dispersion intensity of nanoparticles in the engine oil.

According to Fig. 3, the zeta potential values for most nanolubricants analyzed in this study were greater than 60 mV following a 14-day synthesis period. Based on the classification presented in Table 2, this indicates that the MoS_2_–hBN nanoparticles in the engine oil demonstrated excellent stability. MoS_2_–hBN hybrid nanoparticles possess a high density of negative surface charges due to the dissociation of sulfide ions (S^2−^), borate ions (BO^3−^), and nitride ions (N^3−^) at the particle surface in the nanolubricant. These negative charges repel each other, creating a strong electrostatic barrier between the particles and preventing agglomeration^38,39^. As a result, MoS_2_ nanoparticles remain well dispersed in the liquid medium, leading to a high Zeta potential value. The high Zeta potential of MoS_2_–hBN hybrid nanolubricants has several advantages, including improved stability, reduced friction, and enhanced wear resistance. Additionally, the high Zeta potential helps prevent particle aggregation, which could otherwise lead to clogging of lubrication channels or cause abrasive wear^36,40^. Visual observation tests were conducted to assess the stability of the nanolubricant formulation after the introduction of nanoparticles and over 14 days, as shown in Fig. 3b,c. Notably, no discernible change or sedimentation was observed during the visual inspection. This indicates that the nanoparticles were effectively dispersed and well-incorporated within the nanolubricant matrix, exhibiting excellent stability over the specified duration. The absence of visual alterations or settling further affirms the suitability of the formulation for potential applications in tribological systems. These findings contribute evidence of the successful integration and long-term stability of nanoparticles within the nanolubricant, paving the way for enhanced tribological performance and extended service life^41,42^.

**Table 2:** Zeta potential (mV) values^43^.

Zeta potential (mV)	Stability behaviour of the colloid
From 0 to ± 5	Rapid coagulation or flocculation
From ± 10 to ± 30	Incipient instability
From ± 30 to ± 40	Moderate stability
From ± 40 to ± 60	Good stability
More than ± 61	Excellent stability

**Figure 4:**
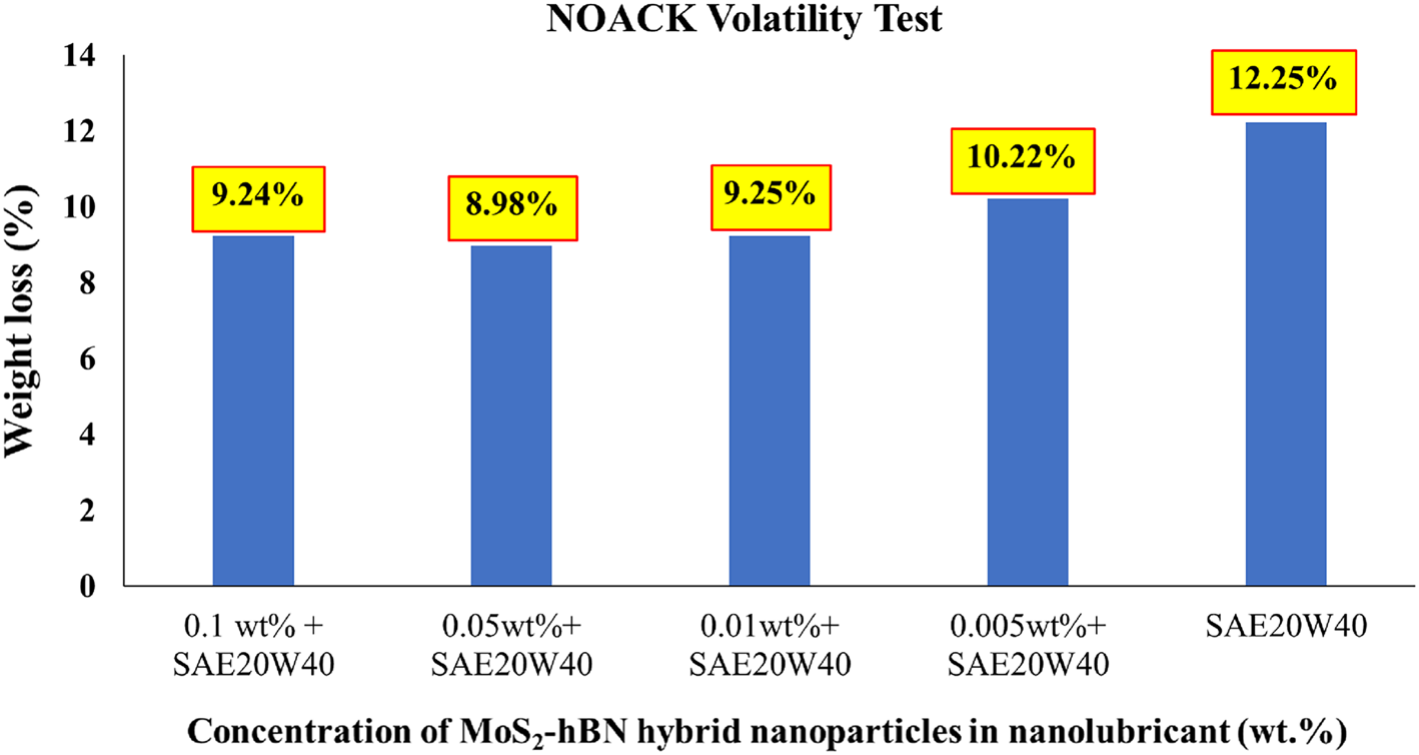
The NOACK Volatility test values of nanolubricant with different concentrations MoS_2_–hBN hybrid nanoparticle.

The loss of engine oil due to volatilization occurs as the temperature rises during engine operation. This is particularly important for engines which operate at higher temperatures. High-performance engines, in particular, generate more heat, which can cause oil to evaporate more quickly. If the oil evaporates too quickly, it can’t provide proper lubrication, leading to engine damage and decreased performance. Noack Volatility Test based on ASTM D5800 is a standardized test method used to measure the evaporation loss of lubricating oils and lubricants. Figure 4 shows the Noack volatility analysis of nanolubricant with various concentrations of MoS_2_–hBN hybrid nanoparticles. The base oil showed an evaporative loss of 12.25%, while all MoS_2_–hBN based nanolubricant showed lower evaporative loss ranging from 8.98 to 10.22%. The addition of the nanocomposite in diesel-based engine oil improves the Noack volatility test findings. The nanocomposite comprises MoS_2_ and hBN, both of which have high thermal stability and can withstand high temperatures without breaking down or evaporating. As a result, including these elements in the nanocomposite helps reduce the volatilization of lighter hydrocarbon components within the oil, resulting in lower total evaporation losses during high-temperature operations. Furthermore, the MoS_2_–hBN nanocomposite has improved boundary lubrication capabilities, which is especially useful under severe pressure circumstances when engine components come into direct metal-to-metal contact^44^. The comprehensive analysis indicated that at a concentration of 0.05 wt%, the nanocomposite exhibited the lowest weight loss during the test. As the concentration increased to 0.1 wt%, a further decrease in weight loss was observed. These findings suggest that the optimal concentration for minimizing weight loss is 0.05 wt%. Deviating from this concentration, both higher and lower levels resulted in increased weight loss. These observations underscore the significance of precisely controlling the concentration of the MoS_2_–hBN nanocomposite in the engine oil to achieve the desired reduction in weight loss during the Noack volatility test. Incorporating these nanoparticles in nanolubricants can enhance the thermal stability of the lubricant, which can avoid the formation of sludge deposition and reduce the tendency of the lubricant to vaporize during the Noack analysis^45^.

(c) Tribological analysis of MoS_2_–hBN hybrid nanolubricant

**Figure 5:**
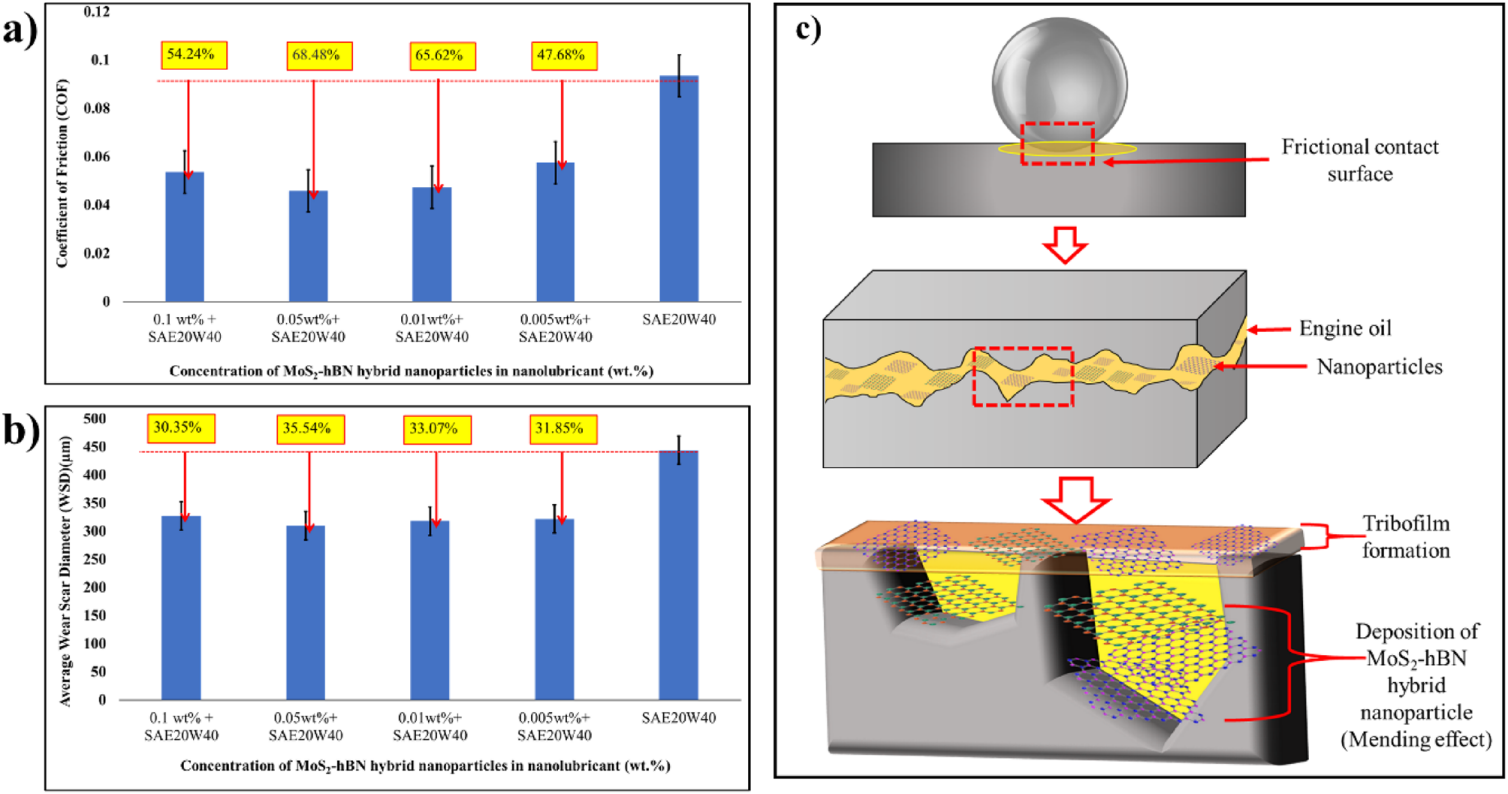
(**a**) COF (**b**) the average WSD of MoS_2_–hBN hybrid nanolubricant (**c**) shematic diagram of lubriacation mechanism of MoS_2_–hBN hybrid nanolubricant.

Figure 5a shows the COF of the nanolubricant at various concentrations of MoS_2_–hBN hybrid nanoparticles in the engine oil. The results showed that the COF of the base oil (pure SAE20W40) was 0.0946. In comparison, the COF reduced to 54.24%, 68.48%, 65.62%, and 47.68% for 0.1 wt%, 0.05 wt%, 0.01 wt%, and 0.005 wt% of the hybrid nanoparticles, respectively. The reduction in COF was attributed to the formation of a boundary film or tribofilm^46–48^. When the MoS_2_–hBN nanoparticles were introduced to the surfaces in contact, they underwent shear deformation and broke down into smaller platelets. These platelets adhered to the surfaces and formed a thin film or layer, which acted as a protective boundary film between the surfaces, reducing the direct contact between them and the coefficient of friction^49,50^. The platelets in the boundary film had low shear strength, allowing them to slide easily against each other and further reduce friction. However, when the concentration of nanoparticles exceeded 0.05 wt%, the COF increased due to agglomeration. Agglomerated nanoparticles can form hard particles that act as abrasives, causing increased surface wear. The roughness created by agglomerated nanoparticles can also increase friction. The formation of a stable and uniform boundary film on the surfaces is essential to reduce friction and wear effectively. The tribological performance of the MoS_2_–hBN hybrid nanolubricant depended on the concentration of nanoparticles. Optimal performance was achieved at a concentration of 0.05 wt%, which provided the best balance between reducing friction and preventing agglomeration. Therefore, it is crucial to control the concentration of nanoparticles to ensure the optimal performance of the nanolubricant.

Figure 5b shows the wear scar diameters (WSD) of hybrid MoS_2_–hBN nanoparticles in SAE20W40 diesel based engine oil using an optical profilometer. The WSD of pure SAE20W40 without the addition of hybrid nanoparticles was 444 µm. It was observed that WSD of the nanolubricant reduced up to 35.54% with the addition of 0.05 wt% MoS_2_–hBN. This is due to the establishment of a thin film, as mentioned earlier. This film acts as a barrier that separates the surfaces and reduces the friction and wear between them^51,52^. The nanoparticles in the film also absorb and distribute the load evenly across the surfaces, reducing localized wear. MoS_2_ and hBN nanoparticles are highly resistant to high temperatures and pressures, making them effective lubricants for applications with extreme conditions. The strong intermolecular forces between the nanoparticles allow them to adhere strongly to the surfaces they are meant to protect, reducing the likelihood of them being displaced or removed during operation. The layered structure of MoS_2_ and hBN also allows them to slide easily against each other, further reducing the wear on the surfaces. The sliding motion helps to distribute the load evenly across the surfaces, reducing localized wear and the formation of wear scars^53^. Figure 5c shows the graphical illustration of the working mechanism that is responsible for the tribological enhancement.

**Figure 6:**
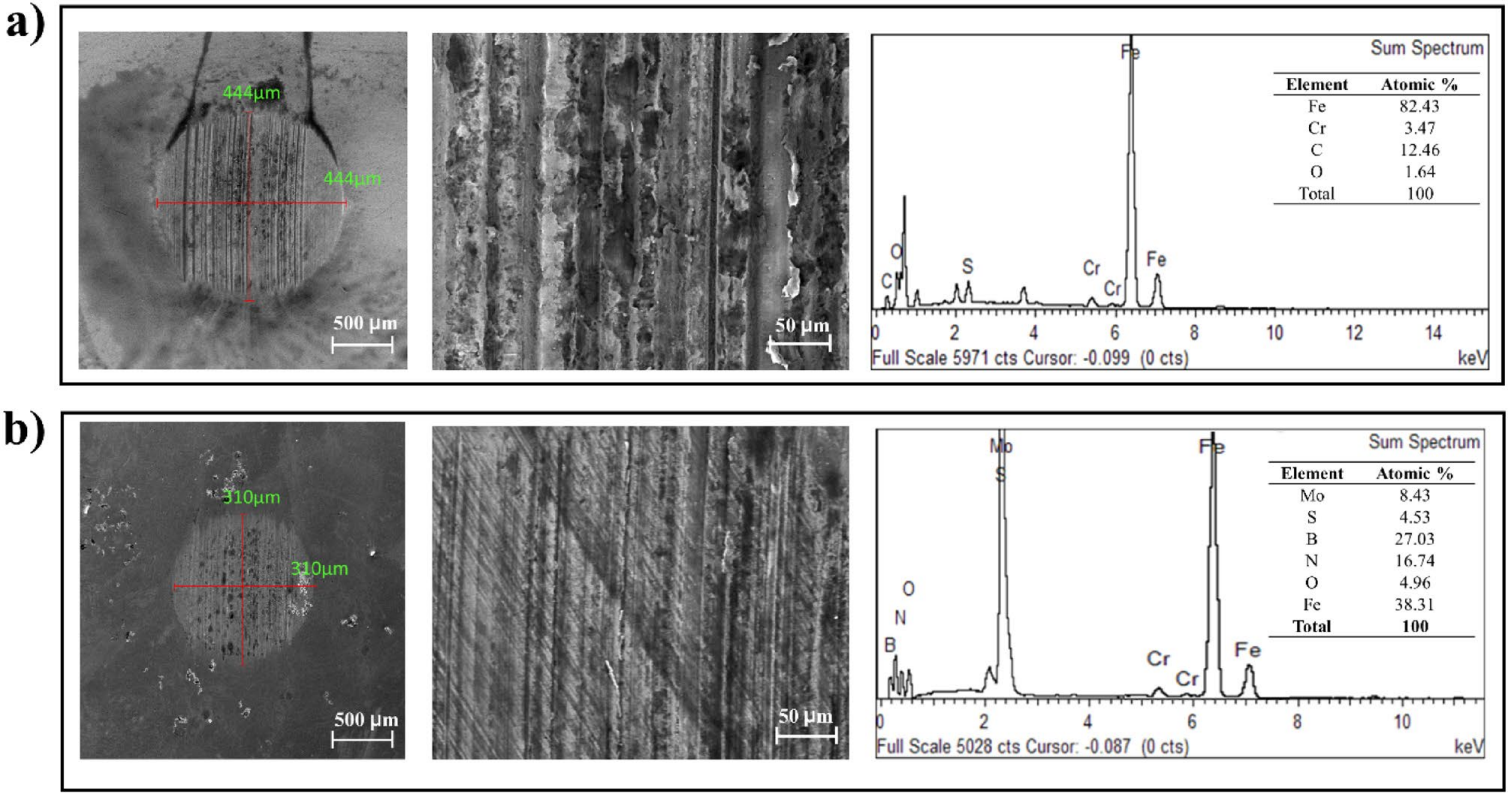
FESEM image of wear scar and corresponding EDX elemental spectrum and atomic distribution of (**a**) of SAE20W40 (**b**) SAE20W40 with 0.05 wt% hybrid MoS_2_–hBN nanoparticle.

FESEM images of wear scars created on a ball bearing during tribological testing are depicted in Fig. 6a,b. A significant difference can be observed between the wear scar created on the steel ball bearing with a base oil and the wear surface of the ball bearing tested with nanolubricant containing 0.05 wt% hybrid MoS_2_–hBN nanoparticles, as indicated by the FESEM images of the wear scar in Fig. 6c. The wear scar on the steel ball bearing with base oil is considerably deeper than that on the ball bearing tested with nanolubricant containing 0.05 wt% hybrid MoS_2_–hBN nanoparticles. It is possible that the deep furrows were reduced with the addition of nanoparticles due to the mending effect of the hybrid MoS_2_–hBN nanoparticles. The mending effect is where the nanoparticle is deposited in the micro-cracks and defects of the mating surfaces, thereby creating a smoother and more uniform surface^54^. Combining MoS_2_ and hBN in a nanocomposite form offers synergistic benefits for reducing the average wear scar diameter observed during tribological testing. MoS_2_ nanoparticles possess exceptional load-carrying capacity and boundary lubrication properties, effectively mitigating metal-to-metal contact and wear. These nanoparticles form a robust, low-friction coating on the sliding surfaces, reducing friction and minimizing wear^48,51,55^. Also, hBN nanoparticles significantly contribute to forming a stable lubricating film, further reducing friction and wear^17,18^. The combined effect of MoS_2_ and hBN additives in the nanocomposite formulation enhances the anti-wear properties of the engine oil, resulting in a reduction in the average wear scar diameter. This synergistic effect is attributed to MoS_2_’s load-bearing capabilities and hBN’s lubricating film formation, which collectively contribute to the superior performance of the nanocomposite in tribological applications. These findings provide valuable insights into the development of advanced lubricant additives and their potential application in improving the durability and efficiency of diesel-based engine oils. These findings can be confirmed from the EDX elemental spectrum where Mo, S, B and N elements were detected on the scar of the ball bearing tested with base oil added with 0.05 wt% hybrid MoS_2_–hBN and the elements of the nanoparticles were not detected on the scar of the ball bearing tested with the base oil (Fig. 6b).

(d) Oxidation and thermal conductivity analysis of MoS_2_–hBN hybrid nanolubricant

**Figure 7:**
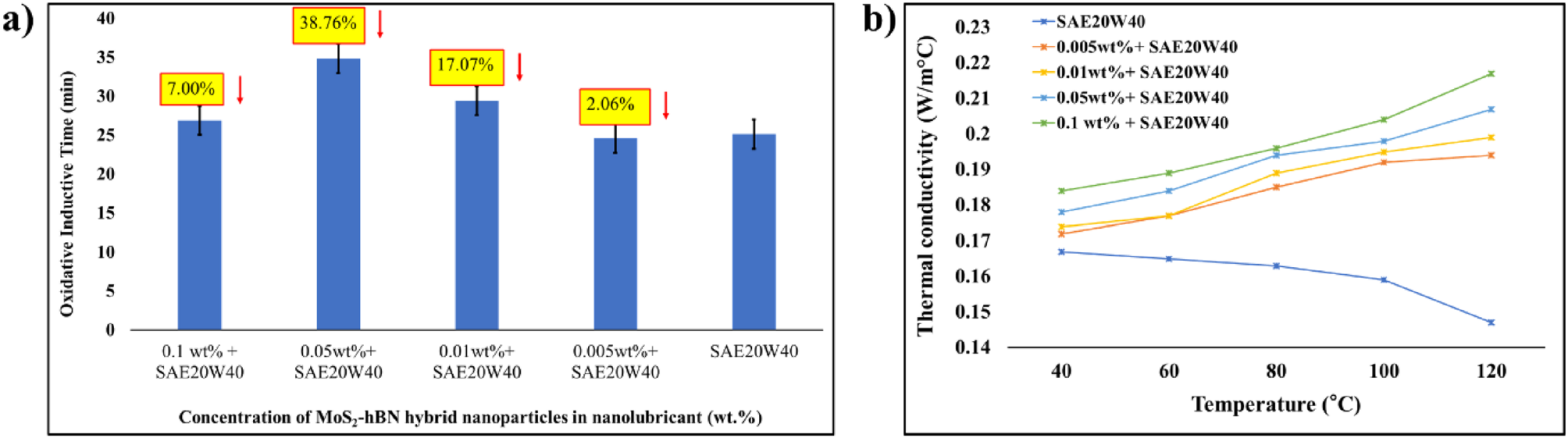
(**a**) OIT analysis and (**b**) thermal conductivity of hybrid MoS_2_–hBN nanolubricant.

Oxidation is a chemical process that occurs when oxygen reacts with hydrocarbons present in engine oil. This process can give rise to a variety of harmful byproducts, such as sludge, varnish, and acids, which can negatively impact the vehicle’s performance and reliability. To assess a lubricant’s resistance to oxidation, an oxidation induction time (OIT) analysis is performed. OIT is the time taken for the lubricant to undergo oxidation under controlled conditions, and the higher the OIT, the more resistant the lubricant is to oxidation. Figure 7a illustrates the OIT of nanolubricant with various concentrations of MoS_2_–hBN hybrid nanoparticles in engine oil. It was noted that the OIT of the nanolubricant increased by up to 38.76% with the addition of 0.05 wt% MoS_2_–hBN hybrid nanoparticles. This is due to the exceptional anti-oxidant properties of MoS_2_ and hBN, which can enhance the oxidative stability of the engine oil. When the nanoparticles are added to the engine oil, they accumulate at the surface of the metal components and form a protective barrier that prevents further oxidation. The nanoparticles form a protective layer on the surface of the metal components, which helps to reduce friction and wear, and also inhibits the formation of free radicals and other oxidizing species^56,57^. This barrier also prevents the formation of harmful deposits, such as sludge and varnish, which can accumulate in the engine and impede the flow of oil to crucial components. By inhibiting the free radical formation and preventing harmful deposit formation, the OIT of the engine oil increases, which means that the oil remains stable and effective for a longer period of time. This can result in improved engine performance and increased component lifespan^58^.

Thermal conductivity is a crucial property of engine oil as it plays a significant role in the oil’s ability to dissipate heat from the engine components. When an engine is in operation, it generates an enormous amount of heat, and it is the oil’s responsibility to carry this heat away from the engine to avoid overheating and prevent component failure^59^. In this study, the laser flash method was employed to determine the thermal conductivity of the nanolubricants formulated with hybrid MoS_2_–hBN nanoparticles. Figure 7b depicts that the addition of hybrid MoS_2_–hBN nanoparticles in SAE20W40 based diesel oil significantly increased the thermal conductivity of the engine oil. For instance, adding 0.05 wt% MoS_2_–hBN hybrid nanoparticle increased thermal conductivity by 28.30% (at 100 °C). The incorporation of MoS_2_–hBN hybrid nanoparticles into engine oil enhances its thermal conductivity by creating more pathways for heat transfer within the oil. These hybrid nanoparticles are known to possess high thermal conductivity properties, as MoS_2_ can withstand temperatures up to 400 °C, while hBN can remain thermally stable at 1000 °C^60,61^. When these nanoparticles are added to engine oil, they create an interconnected network of pathways within the oil, facilitating heat transfer from the engine components to the oil. As a result, the oil acts as an effective heat sink, enhancing engine performance and preventing overheating. Additionally, the nanoparticles can also improve the heat transfer properties of the engine components themselves. As the nanoparticles accumulate on the surface of the metal components, they form a thin layer that can improve the thermal contact between the components and the surrounding oil. This can help reduce the temperature gradient between the components, reducing the risk of component failure and preventing thermal stresses^48,62^.

## Conclusion

This study investigated the effect of hybrid MoS_2_–hBN nanoparticles on the tribological, oxidation and thermal conductivity behavior of SAE20W40 diesel-based engine oil. The nanoparticles were synthesized using an advanced microwave platform, significantly reducing the synthesis time and energy consumption. The MoS_2_ nanoparticles grew uniformly on the surface of hBN nanoparticles due to the interaction between their functional groups. The addition of 0.05 wt% hybrid MoS_2_–hBN nanoparticles in the engine oil significantly reduced friction coefficient and average wear scar diameter by 68.48% and 35.54%, respectively, compared to the base oil. Moreover, the hybrid nanoparticles significantly enhanced oxidation and thermal conductivity by 38.76% and 28.30% (at 100 °C), respectively. The improved tribological behaviour of the nanolubricant can be attributed to the formation of a protective layer on the surface of metal components, reducing friction and wear and inhibiting the formation of harmful deposits such as sludge and varnish. The nanoparticles also formed a network of interconnected pathways within the oil, allowing for more effective heat transfer from the engine components to the oil. These improved heat transfer properties can help prevent overheating and reduce the risk of component failure. The synthesis of hybrid MoS_2_–hBN nanoparticles using an advanced microwave platform has shown excellent improvement in the tribological, oxidation, and thermal conductivity properties of SAE20W40 diesel-based engine oil.

### Experimental details

- Materials.

For the experiment, all chemicals purchased were of analytical grade and used as received without any further modifications. The chemicals used for the synthesis of hybrid MoS_2_–hBN nanoparticles were ammonium molybdate tetrahydrate ((NH_4_)6Mo_7_O_24_·4H_2_O) obtained from Fisher Chemicals in Chicago, USA, thiourea (SC(NH_2_)_2_), and hexagonal boron nitride (hBN) purchased from R&M Chemicals in Dundee, UK. The lubricant oil utilized in the experiment was diesel engine oil with API SAE20W40 CD/SE GL-4.

(b) Synthesis of hybrid MoS_2_–hBN nanoparticle.

**Figure 8:**
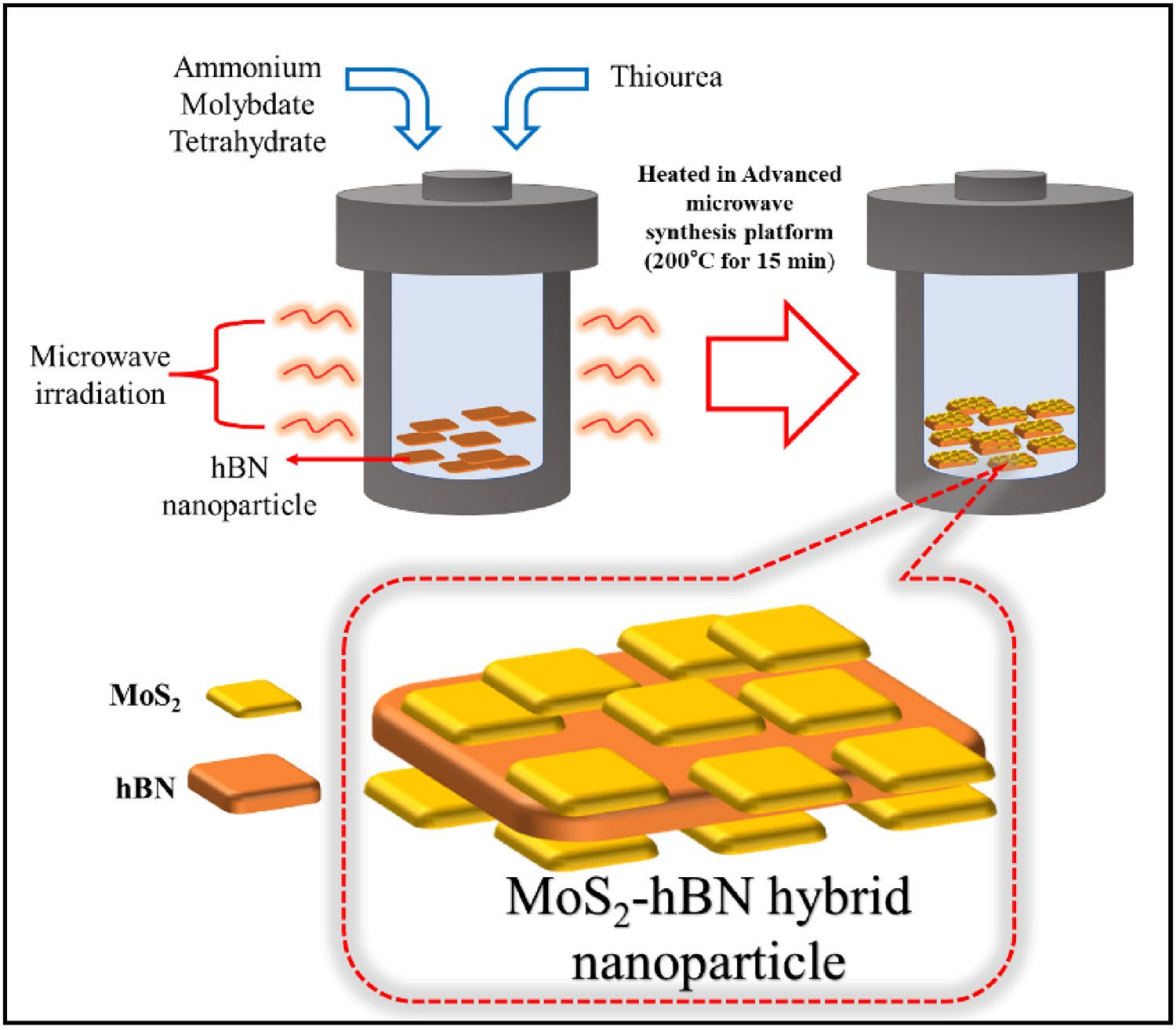
The graphical illustration of synthesizing method of hybrid MoS_2_–hBN nanoparticle.

The synthesis of hybrid MoS_2_–hBN nanoparticles was carried using an advanced microwave hydrothermal synthesis platform. Initially, a solution was prepared by gradually adding 3.7 g of ammonium molybdate tetrahydrate and 6.85 g of thiourea to 105 ml of deionized water while stirring for 30 min to ensure a uniform mixture. Subsequently, 1 g of hBN powder was added, and the mixture was sonicated for an additional 30 min. The resulting mixture was then transferred into a hydrothermal reactor and placed within the microwave synthesis platform (Milestone, flexiWAVE, Italy). The temperature was set to 200°C, and the reaction was allowed to proceed for 15 min. After the reaction, the mixture was allowed to cool naturally to room temperature (26 °C). The sample was then centrifuged and washed using distilled water and ethanol before being freeze-dried. Figure 8 shows the synthesis method of the hybrid MoS_2_–hBN nanoparticles.

(c) Formulation of hybrid MoS_2_–hBN based nanolubricant.

Previous experimental data was utilized to optimize the weight percentage of the MoS_2_ and hBN nanocomposite. Tribological performance showed a decline beyond 0.1 wt and below 0.005 wt. Hence, weight percentages of 0.005 wt%, 0.01 wt%, 0.05 wt%, and 0.1 wt% were selected for further investigation to strike a balance between tribological properties and performance^63^. This range offers valuable insights for optimizing the nanocomposite formulation and advancing its application in lubrication systems. In order to create the nanolubricant, various concentrations of hybrid MoS_2_–hBN nanoparticles were dispersed in 100 ml of SAE 20W40 engine oil, including 0.005 wt%, 0.01 wt%, 0.05 wt%, and 0.1 wt%. The blending process involved homogenizing the mixture for 10 min utilizing a high-shear lab mixer. Subsequently, to achieve a consistent and non-agglomerated dispersion of the nanoparticles within the base oil, the samples underwent ultrasonication in an ultrasonic bath for 30 min.

(d) Characterizations.

Various characterization and analysis techniques were used to study the properties of hybrid MoS_2_–hBN nanoparticles and their performance as a nanolubricant. The size, morphology, and elemental compositions of the nanoparticles were determined by Field Emission Scanning Electron Microscopy and Energy-Dispersive X-ray spectroscopy (FESEM and EDX). The crystallinity and phase structure of the samples were studied using X-ray diffraction (XRD) with Cu Kα radiation (U = 45 kV, I = 27 mA, and λ = 1.54 nm), while the molecular interactions were analyzed by Raman spectroscopy excited with a 532 nm laser. The density, kinematic viscosity, and viscosity index of the samples were measured using a viscometer (Viscometer SWM 3000), and Zetasizer determined the dispersion stability. The Noack volatility test was conducted using Thermogravimetric Analysis (TGA).

Tribological analysis was performed using a four-ball tribotester (Ducom TR-30L) to determine the coefficient of friction (COF) and average wear scar diameter (WSD) of hybrid MoS_2_–hBN -based nanolubricant at various nanoparticle concentrations. Carbon chromium steel balls were used, and the rotational speed, applied load, time, and temperature were 12,000 rpm, 392.5 N, 3600 s, and 75 °C, respectively, in accordance with ASTM 4172-94. The wear scar images on the metal balls were studied by FESEM and EDX.

Oxidation analysis was conducted using High-Pressure Differential Scanning Calorimeter (HP-DSC) to determine the oxidation induction time (OIT) of MoS_2_–hBN based nanolubricant with various nanoparticle concentrations. The procedure was conducted at 500 psi, isothermal temperature of 200 °C, with a flow rate of 50 ml/min and ramping rate of 10 °C/min.The thermal conductivity of the MoS_2_–hBN based nanolubricant with various nanoparticle concentrations was evaluated using laser flash analysis (LFA HyperFlash). The sample was filled in the sample ring, and the upper and lower sealing discs were sprayed with graphite to promote black body absorption. The sample was heated from room temperature to 120 °C at a rate of 10 °C/min in a nitrogen environment.

## Data Availability

All data generated or analyzed during this study are included in this published article.
